# *AutoLEI*: A step towards automatic 3D ED data processing

**DOI:** 10.1107/S2052252525011108

**Published:** 2026-01-01

**Authors:** Shitao Wu, Yanhang Ma

**Affiliations:** a Shanghai Key Laboratory of High-resolution Electron Microscopy & State Key Laboratory of Quantum Functional Materials, School of Physical Science and Technology ShanghaiTech University Shanghai 201210 People’s Republic of China

**Keywords:** 3D electron diffraction, 3D ED, microED, *XDS*, automation

## Abstract

A user-friendly interface has been developed for *XDS*, enabling efficient real-time and batch processing of 3D ED data to accelerate structure determination.

Three-dimensional electron diffraction (3D ED), also known as microcrystal electron diffraction (microED), is an emerging crystallographic methodology that facilitates atomic-resolution structural determination of nano- and microcrystalline specimens through transmission electron microscopy (TEM) (Croat *et al.*, 1984 ▸; Gruene & Mugnaioli, 2021 ▸; Huang *et al.*, 2021 ▸; Mu *et al.*, 2021 ▸). Unlike conventional techniques such as X-ray diffraction (XRD) or neutron diffraction – which need relatively large single crystals – 3D ED can analyze crystallites that are only several hundred nanometres or smaller, rendering it indispensable for investigating materials where growing large crystals is difficult. This capability stems from the strong electron–matter interactions inherent to the technique, which enable data acquisition from sub-micrometre areas. In recent years, 3D ED/microED has demonstrated considerable potential in materials science and structural biology, particularly for nanocrystalline systems (Hu *et al.*, 2025 ▸; Ling *et al.*, 2022 ▸; Vlahakis *et al.*, 2025 ▸). Over the decades of rapid development, the widespread adoption of high-efficiency data acquisition protocols has led to the generation of extensive datasets during experimental procedures (Luo *et al.*, 2023 ▸). Concurrently, traditional manual processing approaches, which are inherently inefficient and insufficient for managing massive data analyses and real-time feedback demands, result in a critical bottleneck. This challenge necessitates the development of specialized software capable of robust, automated processing of these massive datasets.

*XDS* (*X-ray Detector Software*) (Kabsch, 2010 ▸) is a widely used software package for processing diffraction data obtained from single-crystal X-ray diffraction (XRD) or electron diffraction (ED) experiments. Originally designed for X-ray crystallography, it has also been adapted for three-dimensional electron diffraction (3D ED/microED) due to its robust indexing, integration and scaling capabilities. However, its reliance on manual input file preparation and inadequate automation limit its efficacy in high-throughput scenarios.

In the current issue of *IUCrJ*, Wang *et al.* (2026 ▸) provide their solution by developing an *XDS*-based pipeline, named *AutoLEI*, to overcome the low automation level and difficulty in batch processing of 3D ED data (see Fig. 1 ▸). The *AutoLEI* software integrates the *XDS* engine with a graphical user interface (GUI), providing an efficient and user-friendly solution for real-time and batch processing of 3D ED/microED data. This innovation marks a practical improvement in automated processing within this field.

The core strength of *AutoLEI* lies in its clear modular processing workflow and user-friendly interface. Its key technical features include:

(1) *Automation and modularization*. *AutoLEI* organizes data processing into six core workflows (*Input*, *XDSRunner*, *CellCorr*, *XDSRefine*, *MergeData*, *Cluster&Output*), each featuring intuitive GUI tabs for friendly operation. For example, *Input* allows users to set up the work directory, instrument parameters and experimental settings. The *XDSRunner* module automatically generates XDS.INP files and executes *XDS*, extracting critical information and metrics like the space group and unit-cell parameters.

(2) *Real-time processing and dynamic feedback*. *AutoLEI*’s real-time processing capability stands out as another key feature. During data collection, the software instantly analyzes individual data quality metrics (such as resolution and signal-to-noise ratio) and generates HKL files in real time. It will also decide whether to include the data when merging datasets based on predefined criteria. Through dynamically updated charts (like resolution and CC_1/2_), users can adjust collection strategies.

(3) *Broad compatibility*. The software supports multiple data formats (SMV, MRC, TIFF) and acquisition systems (*e.g.EPUD*, *Instamatic*), with Python programming interfaces enabling user-defined extensions. Furthermore, *AutoLEI* features specialized optimizations for low-symmetry crystals and beam-sensitive samples.

The paper demonstrates *AutoLEI*’s versatility through four typical cases:

(1) *Tyrosine crystals with high symmetry and stability*. When processing 12 sets of tyrosine data, statistic values were improved after applying *XDSRefine* in *AutoLEI* and one optimal dataset was identified in just 5 minutes (CC_1/2_ reached 98.18%). Furthermore, the space group was deduced as *P*2_1_2_1_2_1_ through an interactive check of the reflection conditions, finally resolving the high-precision structure including hydrogen atoms.

(2) *The MOF SU-100, with low symmetry*. For crystals with low symmetry, a large tilting angle or multiple datasets are required to achieve a high completeness. Fourteen out of 16 datasets with Isa higher than 5 were merged using *AutoLEI*. Further clustering analysis based on the pairwise correlation coefficients of reflection intensities was conducted and a finalized HKL file was obtained by merging 11 datasets. All non-hydrogen atoms could be located and refined anisotropically using the final HKL file, verifying the need for data merging for quality enhancement.

(3). *The MTH1 protein, with electron-beam sensitivity*. For electron-beam sensitive materials, only a small tilting range can be scanned, as their structures will be damaged quickly under electron-beam irradiation. This makes their structural analysis difficult. For 38 small-angle (15–20°) datasets, *AutoLEI*’s *XDSRefine* function identified 28 datasets, ultimately resolving MTH1’s structure at 2.76 Å resolution.

(4) *Triclinic lysozyme (real-time data processing)*. Although lysozyme crystals diffract to high resolution, structural analysis is challenging due to their low (triclinic) symmetry and preferred orientation. The implementation of real-time data processing allows users to decide how to optimize the data collection strategy and to stop when enough data are collected. By combining this with automated data collection, rapid structure solution becomes possible. In this particular case, 71 datasets were collected within 6 h and 56 datasets were merged, giving a high-resolution analysis to 1.1 Å.

Finally, the authors also discuss further developments, such as introducing other data processing engines. This will enable comparative analyses and data integration, and will lower the technical barriers of 3D ED. This aim aligns closely with the materials science and structural biology trend toward high-throughput and automation.

In summary, *AutoLEI* resolves one headache in 3D ED/microED data processing by integrating automated workflows with a GUI, demonstrating unique value in data merging and real-time feedback. It provides a user-friendly interface for non-specialists. With continued development, this software is poised to become a useful tool in electron crystallography.

## Figures and Tables

**Figure 1:**
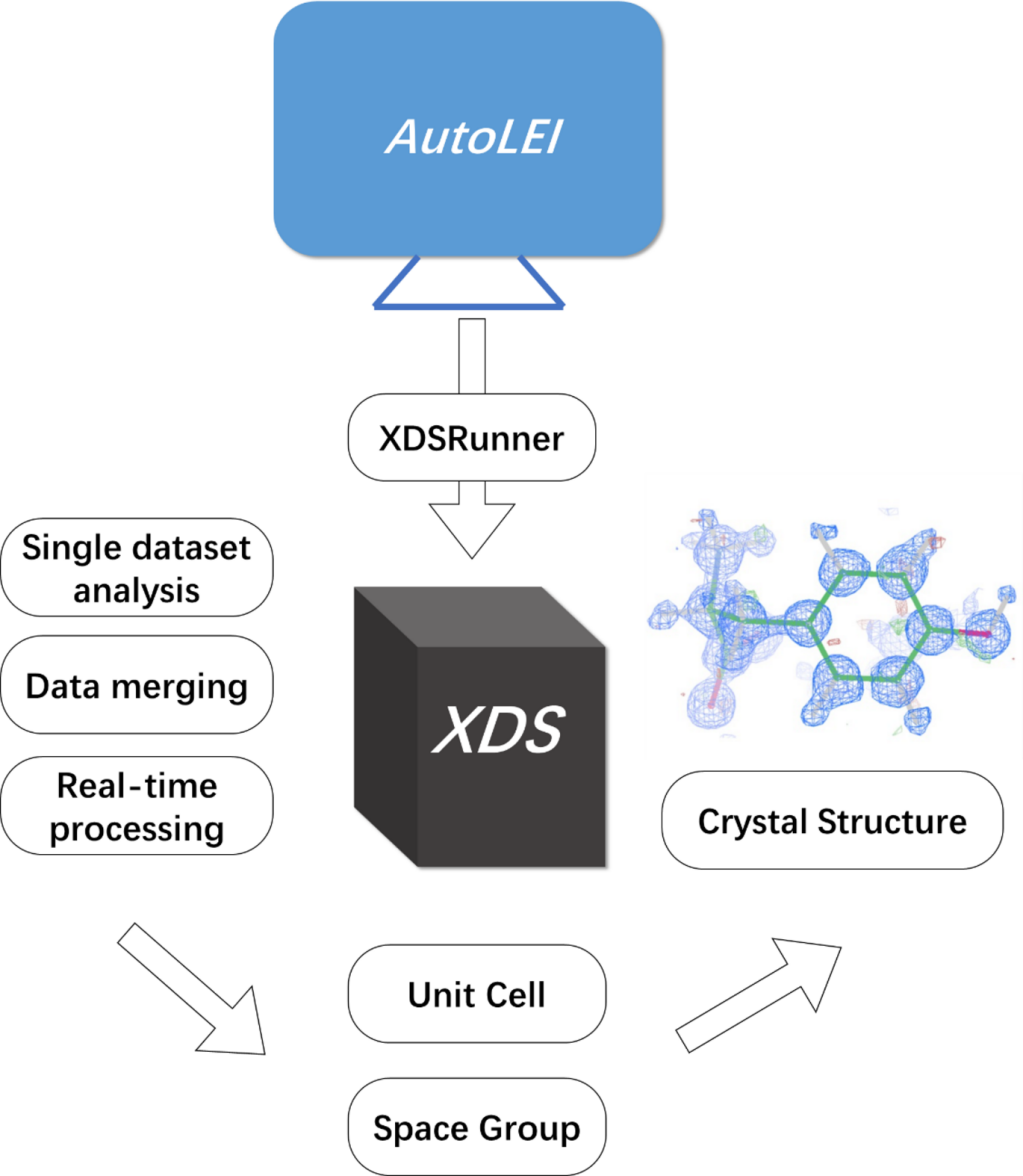
The workflow of *AutoLEI* for single-crystal structural analysis using 3D ED. The image of structure model is reproduced from Wang *et al.* (2026 ▸).

## References

[bb1] Croat, J. J., Herbst, J. F., Lee, R. W. & Pinkerton, F. E. (1984). *Appl. Phys. Lett.* **44**, 148–149.

[bb2] Gruene, T. & Mugnaioli, E. (2021). *Chem. Rev.* **121**, 11823–11834. 10.1021/acs.chemrev.1c00207 PMC8517952 34533919

[bb3] Hu, J., Dong, Z., Chu, C. & Ma, Y. (2025). *Nat. Chem.* **17**, 1819–1825. 10.1038/s41557-025-01950-5 40993250

[bb4] Huang, Z., Grape, E. S., Li, J., Inge, A. K. & Zou, X. (2021). *Coord. Chem. Rev.* **427**, 213583.

[bb5] Kabsch, W. (2010). *Acta Cryst.* D**66**, 125–132. 10.1107/S0907444909047337 PMC2815665 20124692

[bb6] Ling, Y., Sun, T., Guo, L., Si, X., Jiang, Y., Zhang, Q., Chen, Z., Terasaki, O. & Ma, Y. (2022). *Nat. Commun.* **13**, 6625. 10.1038/s41467-022-34237-1 PMC9636419 36333303

[bb7] Luo, Y., Wang, B., Smeets, S., Sun, J., Yang, W. & Zou, X. (2023). *Nat. Chem.* **15**, 483–490. 10.1038/s41557-022-01131-8 PMC10070184 36717616

[bb8] Mu, X., Gillman, C., Nguyen, C. & Gonen, T. (2021). *Annu. Rev. Biochem.* **90**, 431–450. 10.1146/annurev-biochem-081720-020121 PMC9974886 34153215

[bb9] Vlahakis, N. W., Flowers, C. W., Liu, M., Agdanowski, M. P., Johnson, S., Summers, J. A., Jacobs, L. M. C., Keyser, C., Russell, P., Rose, S. L., Orlans, J., Adhami, N., Chen, Y., Sawaya, M. R., Basu, S., de Sanctis, D., Chen, Y., Wakatsuki, S., Nelson, H. M., Loo, J. A., Tang, Y. & Rodriguez, J. A. (2025). *Proc. Natl Acad. Sci. USA* **122**, e2503780122. 10.1073/pnas.2503780122 PMC12337315 40720654

[bb10] Wang, L., Chen, Y., Hutchinson, E. S., Stenmark, P., Hofer, G., Xu, H. & Zou, X. (2026). *IUCrJ* **13**, 105–115. 10.1107/S2052252525010784 PMC12809502 41431443

